# Cordycepin Ameliorates High Fat Diet-Induced Obesity by Modulating Endogenous Metabolism and Gut Microbiota Dysbiosis

**DOI:** 10.3390/nu16172859

**Published:** 2024-08-27

**Authors:** Yifeng Fu, Qiangfeng Wang, Zihan Tang, Gang Liu, Guiping Guan, Jin Lyu

**Affiliations:** 1 Hunan Provincial Engineering Research Center of Applied Microbial Resources Development for Livestock and Poultry, College of Bioscience and Biotechnology, Hunan Agricultural University, Changsha 410128, China; 2 Department of Pathology, The First People’s Hospital of Foshan, Foshan 528000, China

**Keywords:** cordycepin, high-fat diet, fat accumulation, endogenous metabolism, gut microbiota

## Abstract

Background: Numerous metabolic illnesses have obesity as a risk factor. The composition of the gut microbiota and endogenous metabolism are important factors in the onset and progression of obesity. Recent research indicates that cordycepin (CRD), derived from fungi, exhibits anti-inflammatory and antioxidant properties, showing potential in combating obesity. However, further investigation is required to delineate its precise impacts on endogenous metabolism and gut microbiota. Methods: In this work, male C57BL/6J mice were used as models of obesity caused by a high-fat diet (HFD) and given CRD. Mice’s colon, liver, and adipose tissues were stained with H&E. Serum metabolome analysis and 16S rRNA sequencing elucidated the effects of CRD on HFD-induced obese mice and identified potential mediators for its anti-obesity effects. Results: CRD intervention alleviated HFD-induced intestinal inflammation, improved blood glucose levels, and reduced fat accumulation. Furthermore, CRD supplementation demonstrated the ability to modulate endogenous metabolic disorders by regulating the levels of key metabolites, including DL-2-aminooctanoic acid, inositol, and 6-deoxyfagomine. CRD influenced the abundance of important microbiota such as *Parasutterella*, *Alloprevotella*, *Prevotellaceae*_NK3B31_group, *Alistipes*, unclassified_*Clostridia*_vadinBB60_group, and unclassified_*Muribaculaceae*, ultimately leading to the modulation of endogenous metabolism and the amelioration of gut microbiota disorders. Conclusions: According to our research, CRD therapies show promise in regulating fat accumulation and stabilizing blood glucose levels. Furthermore, through the modulation of gut microbiota composition and key metabolites, CRD interventions have the dual capacity to prevent and ameliorate obesity.

## 1. Introduction

In recent decades, obesity has grown to be a serious global public health concern due to its many detrimental repercussions [1]. Metabolic illnesses, including hyperlipidemia [2], type 2 diabetes [3], and fatty liver [4], are significantly correlated with obesity. Genetic factors [5] and unhealthy habits [6] are key contributors to overweight, with the latter reducing energy expenditure and promoting fat accumulation. Furthermore, data point to a connection between overweight and an imbalance in the gut flora [7,8,9,10]. Studies have shown that the Firmicutes/Bacteroidota ratio is elevated in overweight individuals, alongside a higher abundance of obesity-related bacteria in this population [11].

The main goals of the clinical medications now used to treat weight gain are to suppress appetite or prevent the gastrointestinal tract from absorbing fat [12], yet these strategies have little success. Gastrectomy is another approach used for overweight and obese patients [13]; however, these methods pose significant risks to patients. Therefore, research into safe and natural weight management solutions is urgently needed. For instance, cordycepin (CRD) has already contributed to the treatment of obesity. Reduced levels of inflammatory markers, increased helpful bacteria, decreased pathogenic bacteria, and decreased weight gain brought on by a high-fat diet (HFD) were all achieved by CRD [14]. CRD was also able to adjust the abundance of two dominant phyla, Bacteroidetes and Firmicutes, and bring their abundance closer to normal levels in a study by Tang et al. [15]. Additionally, short-chain fatty acid levels rose and gene expression linked to lipid and liver metabolism was regulated after CRD treatment [16]. CRD also reduced fat accumulation and decreased enlarged adipocytes [17]. According to Li et al., CRD can decrease increased fat cells, body weight, and lipid levels in the blood and liver [18]. Research by Qi et al. demonstrated that CRD controls glucose tolerance, metabolism, and prevents weight gain [19]. In summary, CRDs have the superior function of being able to regulate fat accumulation and metabolic levels and improve the gut microbiota.

Edible mushrooms have garnered attention for their potential health benefits [20,21]. CRD, a nucleoside derived from fungi [22], exhibits anti-inflammatory properties, prevents lipid accumulation [23], and enhances energy metabolism [24]. Nonetheless, there is a dearth of research indicating the involvement of CRDs in the control of metabolism, which is examined in this work, as well as the weight loss benefits of these nutrients.

In this study, we initially examined the anti-obesity effects of CRD through obesity phenotyping and histopathology analysis. We then explored CRD’s ability to regulate fat storage, enhance glucose tolerance, and impact metabolism. Additionally, we investigated the impact of CRD on gut microbes using microbial sequencing techniques. Overall, our findings provide fresh perspectives on the weight-reducing properties of CRD.

## 2. Materials and Methods

### 2.1. Materials

**Figure 1:**
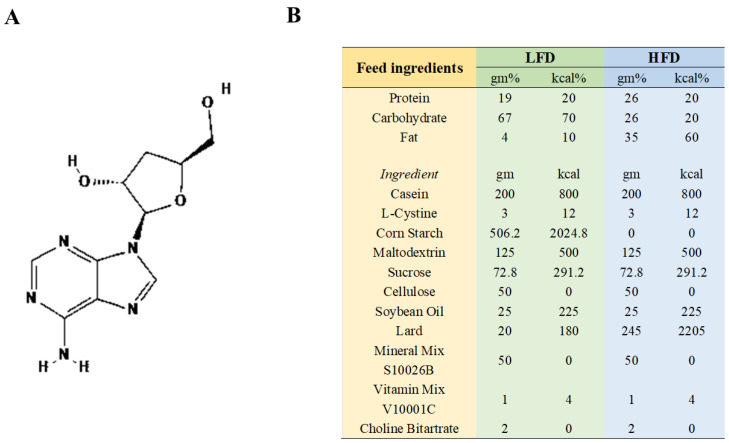
The CRD’s chemical structure and feed content details. (**A**) Chemical structure of CRD; (**B**) Feed composition.

The supplier of CRD (with a 98% purity, CRD is roughly 80% soluble in water) was Meryer (Shanghai) Chemical Technology Co., Ltd., Shanghai, China, The chemical structure of CRD, which was isolated from *Cordyceps sinensis*, is depicted in Figure 1A. The HFD (60 Kcal%, XTHF60) and the corresponding low-fat diet (LFD, 10 Kcal%, XTCON50J) (Figure 1B) were sourced from Jiangsu Xietong Pharmaceutical Bio-engineering Co., Ltd. in Nanjing, China. Low-fat feed and high-fat feed were put in 70–80 g at a time and replaced with fresh feed once every 3–4 days.

### 2.2. Animals and Experimental Design

Forty male C57BL/6J mice, aged 5 weeks and certified as pathogen-free (Certificate of Conformity No.: SCXK (Xiang) 2019-0004), were obtained from Hunan SJA Laboratory Animal Co., Ltd. in Changsha, China. These mice were housed in a carefully controlled environment with a 12 h light-dark cycle, a temperature maintained at 22 ± 1 °C, humidity levels between 50 and 60%, and ad libitum access to food and water. To ensure adequate hydration, water was regularly replenished to allow the mice to drink freely. Approval for this study was obtained from the Biomedical Research Ethics Committee of Hunan Agricultural University (No. 2023-149).

After a one-week acclimation period, the mice were randomly divided into four groups (*n* = 10), with approximately 3–4 mice per cage throughout the 11-week study. The control group was fed an LFD, while another group received the LFD supplemented with CRD in their drinking water (LFD+CRD, 40 mg/kg/day). Furthermore, one group was provided with an HFD to induce obesity, and a separate group received the HFD along with CRD in their drinking water (HFD+CRD, 40 mg/kg/day). The concentration of CRD in the potable water was 0.12 mg/mL. For eleven weeks, mice were fed on their own. The mice’s body weights were recorded weekly. After an 11-week period, the mice underwent an overnight fast before being euthanized by cervical dislocation. Blood was collected from the mouse eye sockets by manual compression of the body. The abdominal skin of the mice was incised upwards using sterilized scissors and secured with pins. Subcutaneous, inguinal, and perinephric fat, as well as liver and colon tissues, were excised and fixed in 4% paraformaldehyde for subsequent staining and observation. Following tissue collection, the intestinal contents were extracted, placed in EP tubes using sterilized forceps, and stored at −80 °C for freezing until further analysis.

### 2.3. Glucose Tolerance Test and Insulin Tolerance Test

Following a 10 h fast, glucose tolerance tests were performed on the mice during Week 10. Intraperitoneal injections of 2 g/kg glucose were administered, and blood samples were collected at 0, 15, 30, 60, and 120 min intervals from the tail tip. Blood glucose levels were measured using test strips from Sannuo, China, and a glucometer [25]. Subsequently, an insulin tolerance test was carried out on the mice after another 10 h fast in Week 11. The mice received intraperitoneal injections of 0.75 U/kg insulin, and blood samples were taken at 0, 15, 30, 60, and 120 min from the tail tip for glucose concentration measurement using a glucometer and test strips from Sannuo, Shenzhen, China [25].

### 2.4. Histopathological Analysis

The fixed tissues, including subcutaneous fatty tissue, inguinal fat, perinephric fat, liver, and colon tissues, were removed from the 4% paraformaldehyde solution. These tissues were then sectioned, embedded in paraffin, stained with hematoxylin and eosin (H&E), and subjected to histopathological analysis using a digital scanner (Wisleap WS-10, Changzhou, China) [26].

### 2.5. Serum Metabolite Analysis

The mice were euthanized by decapitation, and blood samples were collected from their eye sockets. Subsequently, the blood samples were centrifuged at 4 °C and 4000 rpm for 15 min to obtain serum for UPLC-Q-TOF analysis. Chromatographic analysis was performed using an Acquity UPLC HSS T3 C18 column (2.1 × 100 mm, 1.7 μm) with a mobile phase consisting of 0.1% formic acid in H2O (mobile phase A) and 0.1% formic acid in ACN (mobile phase B). Metabolic profiling was carried out using the MassLynx V14.1 platform and the Xevo G2-XS Q-TOF mass spectrometer (Waters, Milford, MA, USA) [27].

### 2.6. Analysis of Intestinal Microorganisms

After extracting genomic DNA from the colon contents, the V3 and V4 regions of the 16S gene were amplified with primers (5′-ACTCCTACGGGGAGGCAGCA-3′ and 5′-GGACTACHVGGGTWTCTAAT-3′). The amplification process was carried out using the Illumina NovaSeq sequencing platform, provided by Biomarker Technologies Co., Ltd., based in Beijing, China.

The raw reads obtained from sequencing were filtered using Trimmomatic v0.33 software. Subsequently, cutadapt 1.9.1 software was employed to identify and remove primer sequences, resulting in clean reads free of primer sequences. The final valid dataset was then generated by denoising, bipartite sequence splicing, and removing chimeric sequences using QIIME [28].

Species diversity was evaluated using the Shannon and Simpson indices through OTU analysis. Following this, PCA analysis and heat maps were created using the oe cloud platform (https://cloud.oebiotech.com/, accessed on 24 June 2024). Furthermore, LDA effect size (LEfSe) analysis was conducted with an LDA threshold exceeding 4 to explore distinct bacterial taxa variations among various groups [29].

### 2.7. Statistical Analysis

After conducting data analysis with IBM SPSS Statistics 25, the results were presented as the mean ± standard deviation (SD). Group comparisons at each time point were assessed through repeated-measures ANOVA, with the data demonstrating a normal distribution. Comparisons between groups were performed using a one-way analysis of variance (ANOVA). Comparisons between the two groups were performed using Graphpad Prism 9 for *t*-tests. Statistical significance was set at *p* < 0.05 to indicate differences.

## 3. Results

### 3.1. Weight Gain and Fat Deposits Are Mitigated by CRD

**Figure 2:**
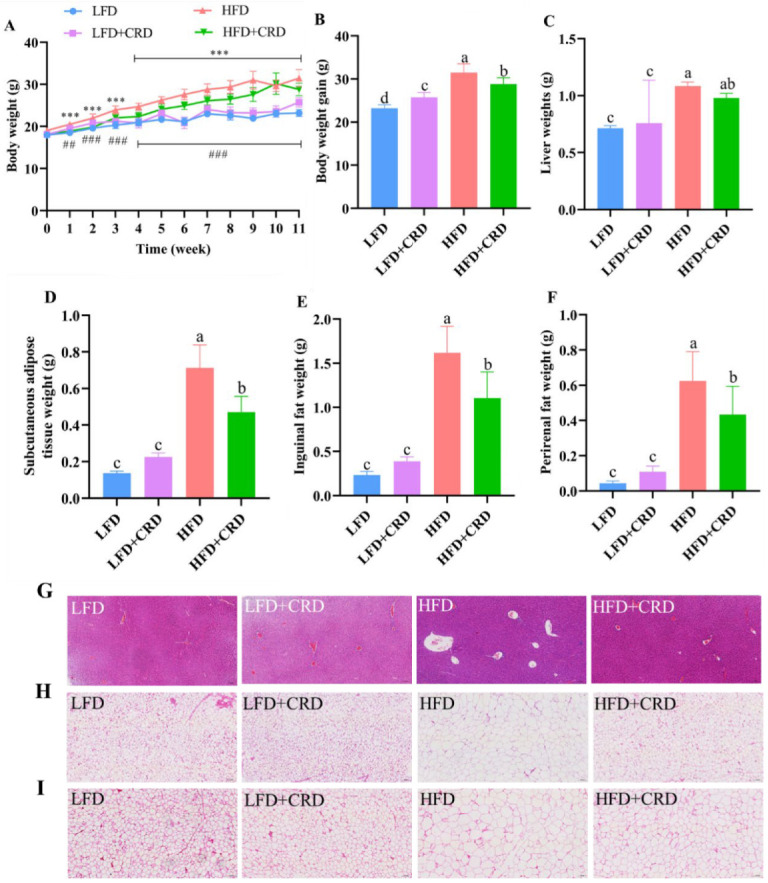
Impact of CRD on fat storage and body weight growth in mice fed a high-fat diet (HFD). (**A**) Body weight changes throughout an 11-week period (*n* = 10); (**B**) Body weight gain (*n* = 10); (**C**) Liver weight; (**D**) Subcutaneous fatty tissue weight; (**E**) Inguinal fat weight; (**F**) Perinephric fat weight; (**G**) Liver representative H&E stained images, scale bar: 100 μm; (**H**) H&E stained images of subcutaneous fatty tissue, scale bar: 100 μm; (**I**) H&E stained images of inguinal fat, scale bar: 100 μm. Data are expressed as the mean ± SD. Different letters on the bar graphs indicate changes that are statistically significant (*p* < 0.05), whereas the same letter indicates no significant difference. *** *p* < 0.001; ^##^ *p* < 0.01; ^###^ *p* < 0.001. Low-fat diet: LFD.

The mice were fed for a total of 11 weeks in four groups: LFD, LFD+CRD, HFD, and HFD+CRD, in order to evaluate the effect of CRD on obese mice. At the conclusion of the trial, mice on HFD gained significantly more weight than those on LFD and LFD+CRD (Figure 2A,B, *p* < 0.05). Notably, the ultimate body weight gain and weight gain were considerably suppressed in mice fed HFD+CRD (Figure 2A,B, *p* < 0.05). Furthermore, our findings revealed significantly elevated liver weight, subcutaneous adipose tissue weight, inguinal fat weight, and perirenal fat weight in the HFD group compared to the LFD group (Figure 2C–F, *p* < 0.05). Conversely, supplementation with CRD led to a significant reduction in subcutaneous fatty tissue, inguinal fat, and perinephric fat weights (Figure 2C–F, *p* < 0.05). Following CRD supplementation, histological analysis of liver vacuoles in HFD-fed mice exhibited a significant reduction (Figure 2G). Moreover, CRD supplementation resulted in a marked decrease in adipocyte enlargement in the subcutaneous fatty tissue and inguinal fat of HFD-fed mice (Figure 2H,I). Collectively, these results indicate that CRD supplementation confers beneficial effects on obesity in mice.

### 3.2. CRD Supplementation Alleviates HFD-Induced Impaired Glucose Tolerance and Intestinal Inflammation

**Figure 3:**
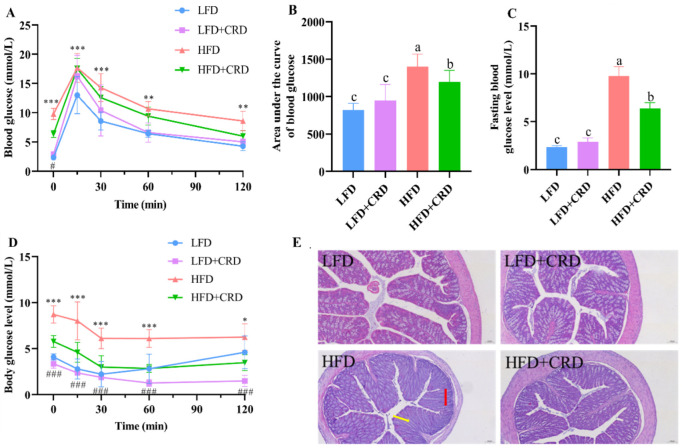
CRD supplementation ameliorates impaired glucose tolerance and intestinal inflammation in obese mice. (**A**) Glucose tolerance test (GTT); (**B**) Area under the GTT curve; (**C**) Fasting blood glucose level; (**D**) Changes in the body’s blood sugar levels after insulin injections; and (**E**) Representative H&E stained images of the colon, scale bar: 100 μm. Data are expressed as the mean ± SD (*n* = 6). The same letters on the bar graphs indicate no significant changes, while different letters indicate statistically significant differences. * *p* < 0.05; ** *p* < 0.01; *** *p* < 0.001; ^###^ *p* < 0.001. “*” indicates LFD group VS HFD group. “#” indicates HFD group vs. HFD+CRD group.

Given the observed reductions in body weight gain and adipocyte hypertrophy in mice following CRD supplementation, we proceeded to investigate the impact of CRD on glucose tolerance, and colonic inflammation. The glucose tolerance test demonstrated a significantly larger area under the curve in mice following the HFD regimen compared to those in the LFD group (Figure 3A,B, *p* < 0.05). In contrast, mice in the HFD+CRD group exhibited a notable reduction in the area under the glucose tolerance curve compared to the HFD group (Figure 3A,B, *p* < 0.05). Moreover, the fasting blood glucose levels of mice in the HFD+CRD group exhibited a significant decrease compared to those in the HFD group (Figure 3C, *p* < 0.05). These results indicate that CRD supplementation effectively mitigated impaired glucose tolerance in mice.

Subsequently, we evaluated the insulin sensitivity of the mice. According to our results, mice in the HFD+CRD group had body glucose levels that were considerably lower than those in the HFD group after insulin was administered. These levels then gradually converged towards those in the normal group (Figure 3D, *p* < 0.05). This difference underscores the enhanced insulin sensitivity of the HFD+CRD group over the HFD mice (Figure 3D, *p* < 0.05), indicating the beneficial impact of CRD supplementation on insulin sensitivity in mice.

Obesity is known to induce low-grade inflammation in the body [30,31]. In our investigation, we delved into the histological integrity of the colon, as depicted in Figure 3E. Hematoxylin and eosin (H&E) staining of the colon illustrated that mice in the LFD group showcased a typical morphological structure of the colon, free from inflammatory infiltration or pathological damage. In contrast, mice in the HFD group exhibited indications of inflammatory infiltration (red arrow) and colonic villi destruction (yellow arrow), both of which were notably improved following CRD intervention (Figure 3E). These results highlight the potential of CRD in alleviating the inflammatory damage induced by an HFD.

### 3.3. CRD Improves Metabolic Disorders Due to HFD

**Figure 4:**
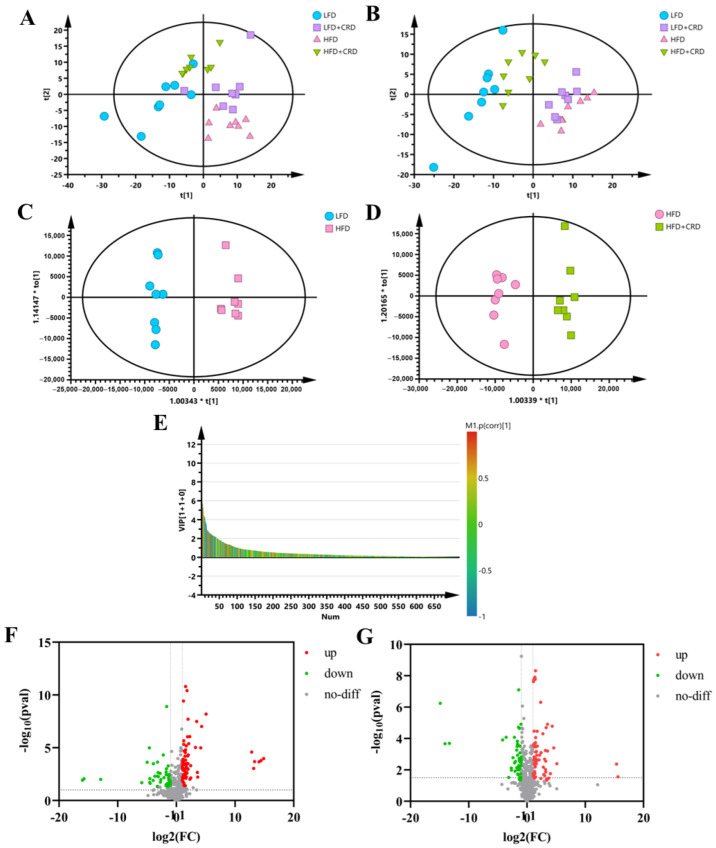
Serum metabolites are altered by CRD supplementation. (**A**) PCA plots of 4 experimental groups in positive-ion mode (*n* = 8); (**B**) PCA plots of 4 experimental groups in negative-ion mode (*n* = 8); (**C**,**D**) OPLS-DA plots in positive-ion mode; (**E**) VIP-plot in positive-ion mode (HFD vs. HFD+CRD); (**F**) Volcano plot of metabolites that changed between LFD and HFD groups; and (**G**) Volcano plot of metabolite changes between the HFD and HFD+CRD groups (*p* < 0.05, |log2FC| > 1).

Given the potential for obesity to induce metabolic disorders in organisms [32,33], we conducted a metabolomic analysis of serum samples from mice. In both positive- and negative-ion modes, the PCA results revealed a notable differentiation between the samples from the LFD and HFD groups. This differentiation suggests that the endogenous metabolites in the HFD group of mice underwent alterations compared to those in the LFD group. Notably, the metabolite profiles of mice in the HFD+CRD group closely resembled those of the LFD group, suggesting that CRD supplementation could mitigate the metabolic disturbances induced by the HFD (Figure 4A,B).

To differentiate between the HFD and LFD groups, as well as the HFD+CRD group, and to identify potential biomarkers, we generated OPLS-DA plots and VIP-plots (Figure 4C–E). These plots clearly demonstrated the segregation of metabolites in the HFD group from the LFD and HFD+CRD groups. Utilizing volcano plots for further investigation of metabolite level changes, it was shown that in the positive-ion mode (|log2FC| > 1, *p* < 0.05), 89 metabolites were upregulated and 97 metabolites were downregulated in the HFD group relative to the LFD group (Figure 4F). Similarly, in the HFD+CRD group, 73 metabolites were upregulated and 55 metabolites were downregulated (|log2FC| > 1, *p* < 0.05) compared to the HFD group (Figure 4G). A metabolite heat map was created when 60 metabolites were identified using the criteria of VIP > 1 and *p* < 0.05 (Supplementary Figure S1A). In comparison to the LFD group, the HFD group had lower levels of several metabolites, including DL-2-aminooctanoic acid, Gentianadine Esi+1.109, Myo-Inositol, and PC(15:0/22:5(4Z,7Z,10Z,13Z,16Z)) Esi+24.497005. Metabolites such as LacCer(d18:1/14:0) Esi+20.789997 and PC(15:0/22:5(4Z,7Z,10Z,13Z,16Z)) Esi+22.901995 were trending upward. Compared to the HFD group, the intervention in the HFD+CRD group resulted in DL-2-aminooctanoic acid, Myo-Inositol, PI-Cer(d18:0/18:0) Esi+20.799006, PC(22:1(11Z)/14:0) Esi+20.862995, PI(16:0/20:5(5Z,8Z,11Z,14Z,17Z)), PE(15:0/24:1(15Z)) 20.934004, PG(P-20:0/18:3(9Z,12Z,15Z)), and Gentianadine Esi+1.109 levels being upregulated (Supplementary Figure S1A, *p* < 0.05). The metabolite levels were generally changed as a result of the CRD intervention, approaching those of the LFD group.

To explore the impact of CRD on metabolic pathways in mice from the HFD group, we conducted metabolite enrichment analysis on 60 metabolites using MetaboAnalyst 6.0 (https://www.metaboanalyst.ca/, accessed on 30 May 2024). This analysis revealed significant effects on key metabolic pathways, including ascorbate and aldarate metabolism, β-alanine metabolism, galactose metabolism, glutathione metabolism, and inositol phosphate metabolism (Supplementary Figure S1B). Our findings suggest that CRD has the potential to ameliorate the metabolic dysregulation induced by an HFD.

### 3.4. Effects of CRD on the Gut Microbiota

**Figure 5:**
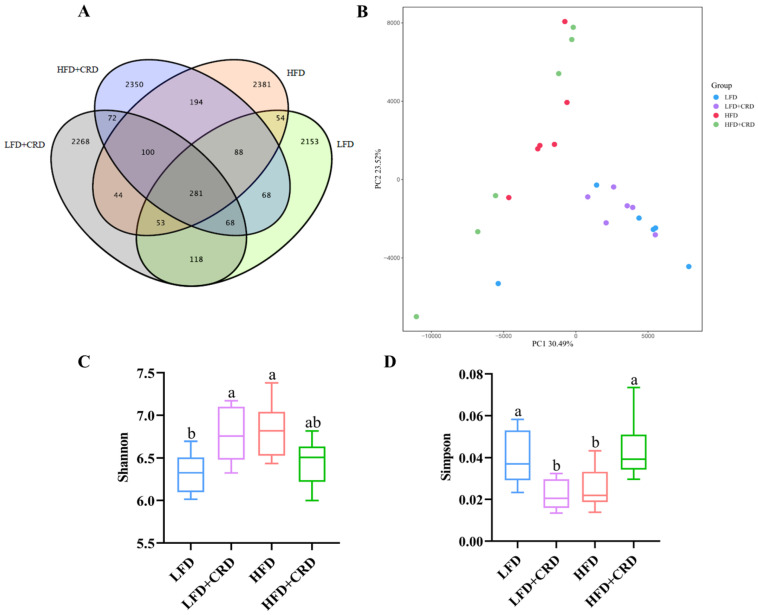

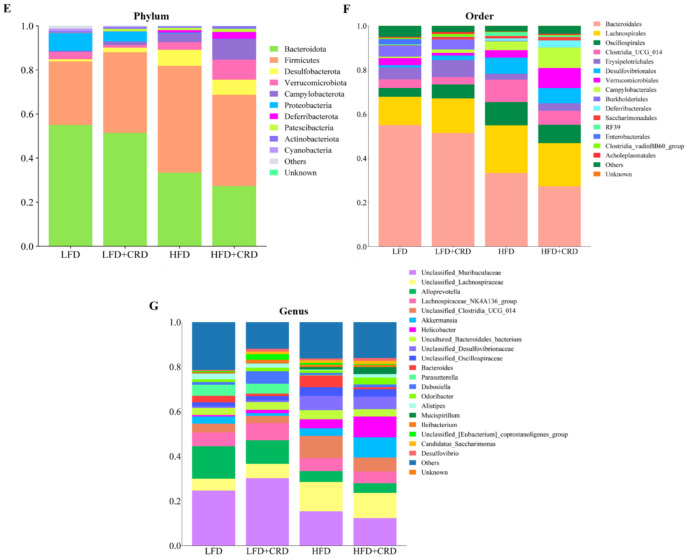
CRD ameliorates HFD-induced gut microbial disorders. (**A**) Wayne plots of the OUT of the intestinal flora of each experimental group; (**B**) PCA analysis plots of each experimental group; (**C**) Shannon’s index; (**D**) Simpson’s index; (**E**) Relative abundance at the level of phylum; (**F**) Relative abundance at the level of order; and (**G**) Relative abundance at the level of genus. Data are expressed as the mean ± SD (*n* = 6). Different letters on the box plots indicate changes that are statistically significant (*p* < 0.05), while the same letter indicates no significant difference.

There is substantial evidence indicating that obesity contributes to gut microbiota dysbiosis [30,34,35,36]. By applying the 97% sequence similarity criterion, we identified 2883, 3004, 3195, and 3221 Operational Taxonomic Units (OTUs) for the LFD, LFD+CRD, HFD, and HFD+CRD groups, respectively, as illustrated in Figure 5A. While the PCA score graph indicated some differences among the groups, these variances were not statistically significant (Figure 5B). Of note is the observation that the HFD group exhibited a higher Shannon index compared to the LFD group, which subsequently decreased post-CRD intervention (Figure 5C, *p* < 0.05). Conversely, the Simpson’s index was lower in the HFD group than in the LFD group but returned to normal levels following the CRD intervention (Figure 5D, *p* < 0.05).

We proceeded to examine the shifts in microbial compositions at the order, genus, and phylum levels, as depicted in Figure 5E–G. Notably, at the order level, Bacteroidales and Lachnospirales emerged as the most predominant among the groups (Figure 5F). Furthermore, at the genus level, the abundance of *Odoribacter*, *Alistipes*, and *Akkermansia* exhibited a decrease in the HFD group and an increase in the HFD+CRD group compared to the LFD group, as shown in Figure 5G, although no significant disparity was observed. Remarkably, the HFD+CRD intervention led to a reduction in the levels of Bacteroides compared to the HFD group (*p* < 0.05).

Following this, we employed LEfSe analysis (LDA > 4) to pinpoint microbial taxa displaying distinctions across the groups. Notably, the CRD intervention instigated alterations in seven microbial classifications, with particularly pronounced changes observed in Rodentibacter and Pasteurellaceae (Supplementary Figure S2A). Furthermore, the concentrations of Firmicutes, Lachnospirales, and Clostridium in the HFD group surpassed those in the control group (Supplementary Figure S2A,B).

**Figure 6:**
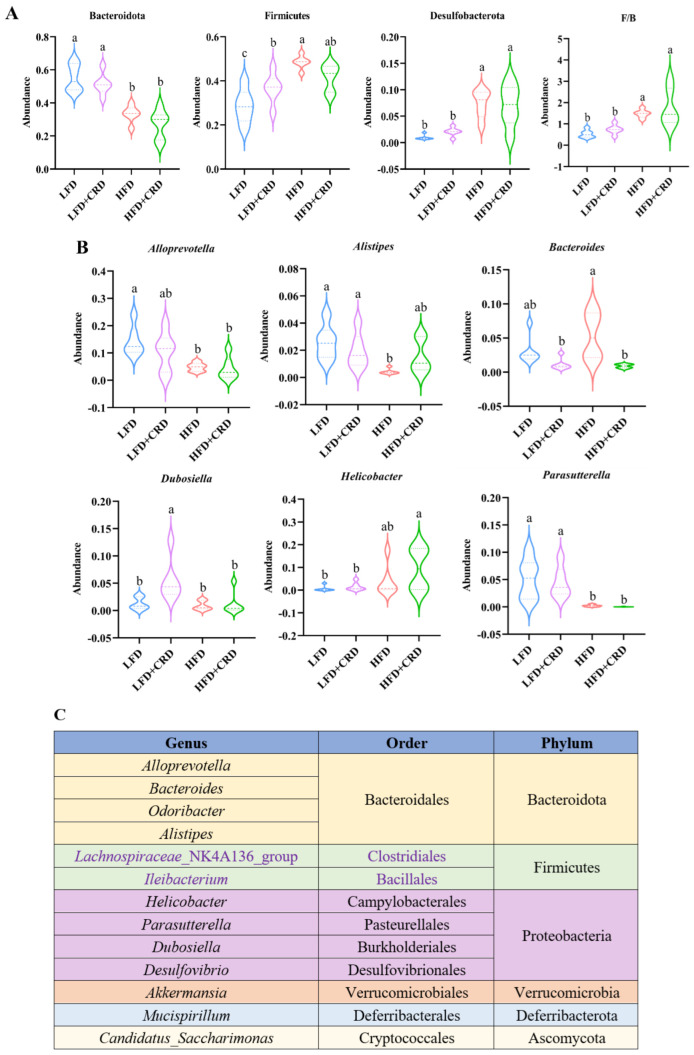
Intervention of CRD resulted in changes in the level of flora in the gut. (**A**) The phylum-level dominant bacteria as well as the Firmicutes to Bacteroidota ratio; (**B**) Highly abundant genera; (**C**) Attributional classification of microorganisms at the genus, order, and phylum level. Data are expressed as the mean ± SD (*n* = 6). On the violin plots, different letters denote statistically significant differences (*p* < 0.05), while the same letter denotes no significant changes.

The top ten bacterial groupings were then examined in terms of both phylum and genus abundance. As depicted in Figure 6A, the HFD group exhibited a lower abundance of the phylum-level Bacteroidota in comparison to the LFD group (*p* < 0.05). While Bacteroidota levels increased in the HFD+CRD group, no significant disparity was observed between this group and the HFD group. Noteworthy differences in the quantities of Firmicutes and Desulfobacterota were evident between the LFD and HFD groups (Figure 6A, *p* < 0.05). Furthermore, at the genus level, the abundance of *Bacteroides* was diminished in the HFD+CRD group relative to the HFD group (Figure 6B, *p* < 0.05). The taxonomic classification of microorganisms at the genus, order, and phylum levels is detailed in Figure 6C. These results underscore the potential of CRD intervention in modulating *Bacteroides* abundance and partially rectifying dysbiotic microbiota.

### 3.5. Relationships between Metabolites and Gut Microorganisms and Markers Associated with Obesity

**Figure 7:**
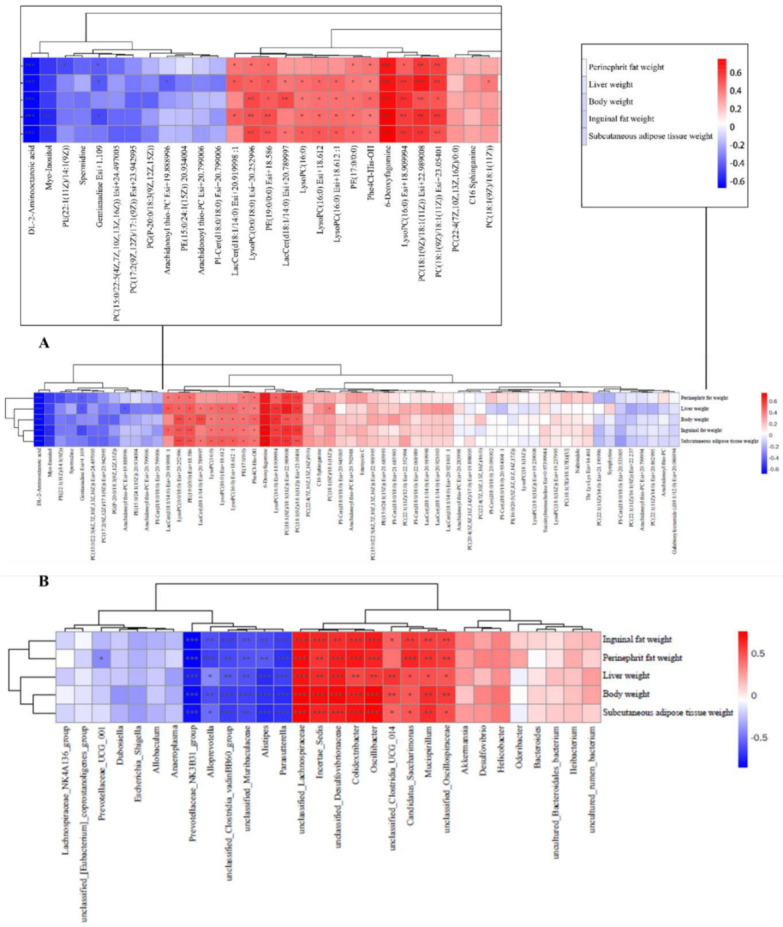
Investigation of the correlation between metabolites and microbes and obesity-related markers. (**A**) Significant correlations are denoted by an “*” in the Spearman correlation analysis of metabolites and obesity-related markers; red indicates a positive connection and blue a negative correlation; (**B**) Significant correlations are denoted by an “*” in the Spearman correlation analysis of microorganisms and obesity-related variables; a positive connection is represented by a blue color, and a negative correlation by a red color. * *p* < 0.05, ** *p* < 0.01, *** *p* < 0.001.

To explore the impact of metabolites and gut microbes on obesity, we conducted Spearman’s correlation analyses between these factors and obesity-related parameters, encompassing inguinal fat weight, perinephric fat weight, liver weight, body weight, and subcutaneous adipose tissue. In Figure 7A, 6-deoxyfagomine, LysoPC(16:0) Esi+18.909994, PC(18:1(9Z)/18:1(11Z)) Esi+22.989008, and PC(18:1(9Z)/18:1(11Z)) Esi+23.05401 exhibited positive correlations with all obesity parameters (inguinal fat weight, perinephric fat weight, liver weight, body weight, subcutaneous adipose tissue weight), indicating their potential contributory role in obesity development. Conversely, DL-2-aminooctanoic acid and Myo-Inositol demonstrated a strong negative correlation with the same obesity parameters, suggesting a potential preventive effect of these compounds on obesity progression.

In addition, the microbial unclassified_Lachnospiraceae, Incertae_Sedis, unclassified_Desulfovibrionaceae, Colidextribacter, Oscillibacter, Candidatus_Saccharimonas, Mucispirillum, and unclassified_Oscillospiraceae, as depicted in Figure 7B, exhibited a significant positive correlation with various obesity-related parameters, including inguinal fat weight, perinephric fat weight, liver weight, body weight, and subcutaneous adipose tissue. These findings highlight their potential involvement in the development of obesity. On the contrary, the presence of Parasutterella, Alistipes, unclassified_Muribaculaceae, unclassified_Clostridia_vadinBB60_group, Alloprevotella, and Prevotellaceae_NK3B31_group demonstrated a notable negative correlation with the same obesity parameters, suggesting a potential role of these bacteria in combating obesity.

## 4. Discussion

Obesity, recognized as a metabolic disorder, is associated with various complications [37,38,39,40]. Recent studies propose that CRD sourced from fungi possesses diverse biological effects and contributes significantly to reducing obesity rates. Thus, in our study, CRD was selected for water intake intervention in HFD-induced obese mice. The impact of CRD was evaluated by comparing changes in body weight, adipocyte size, and blood glucose levels, conducting histological examinations, metabolomics, and 16S rRNA sequencing. Our findings demonstrated that CRD effectively mitigated obesity progression, showcasing anti-obesity properties through enhanced metabolism and the regulation of gut microbiota.

Prior studies have highlighted the hypolipidemic and anti-inflammatory properties of CRD [41,42], along with their positive effects on metabolism and blood glucose regulation [19]. Building on this foundation, our research demonstrated the efficacy of CRD intervention in mitigating weight gain induced by a high-fat diet and reducing adipocyte size, aligning with observations by Jang et al. [43] and Li et al. [18]. Furthermore, CRD exhibited the ability to mitigate hepatocyte vacuolization and diminish adipocyte hypertrophy. Obesity is known to compromise intestinal barrier integrity, leading to intestinal inflammation [44], underscoring the importance of natural interventions in preserving intestinal function. Zhao et al.’s work on obesity highlighted quercetin’s ability to alleviate intestinal inflammation in obese mice [45]. In our investigation, CRD treatment notably ameliorated colonic inflammatory infiltration (Figure 3E), indicating its anti-inflammatory properties. These results suggest that CRD may confer anti-obesity advantages by reducing tissue damage and addressing the obesity phenotype.

Numerous studies have underscored the link between obesity and internal metabolic dysregulation [46,47]. Upon analyzing the serum metabolomics data, we identified DL-2-aminooctanoic acid, inositol, PI-Cer(d18:0/18:0) Esi+20.799006, and PC(22:1(11Z)/14:0) Esi+20.862996 as more prominent in the HFD+CRD group compared to the HFD group. Furthermore, the levels of PI(16:0/20:5(5Z,8Z,11Z,14Z,17Z)) and PE(15:0/24:1(15Z)) 20.934004 were significantly upregulated (Supplementary Figure S1A, *p* < 0.05). Notably, inositol, a crucial polyol essential for cell signaling and ascorbic acid synthesis [48], has demonstrated efficacy in restoring body weight and addressing metabolic disorders in obese individuals [49]. Moreover, inositol has been shown to enhance the expression of key markers in brown adipose tissue, stimulating cellular metabolism and presenting a promising therapeutic approach for obesity treatment [50].

PI-Cer(d18:0/18:0) Esi+20.799006 is ceramide phosphatidylinositol [51]. Ceramide accumulation inhibits insulin signaling [52]. It has been shown that ceramide and phosphatidylinositol levels are elevated in obese patients compared to normal-weight individuals [53]. These reports seem to be contrary to our findings, and the specific effects of CRD on ceramide phosphatidylinositol production need to be further investigated. PC(22:1(11Z)/14:0) Esi+20.862995 belongs to a class of phosphatidylcholines, essential components of animal biofilms [54]. PC(22:1(11Z)/14:0) Esi+20.862995 was found to be higher in the HFD+CRD group than in the HFD group, according to our research. Li et al. demonstrated that phosphatidylcholine promotes brown adipogenesis, offering protection against obesity and metabolic dysfunction [55], aligning with our results.

Meanwhile, PI(16:0/20:5(5Z,8Z,11Z,14Z,17Z)) is phosphatidylinositol, and the phosphatidylinositol-3-kinase (PI3K)/protein kinase B (Akt) signaling pathway has been associated with the maintenance of metabolic homeostasis [56]. According to a study by Wang et al., insulin resistance brought on by a high-fat diet was lessened by activating the PI3K/AKT pathway [57]. In addition, the activation of the PI3K/AKT signaling pathway can trigger autophagy, which can reduce lipid deposition and inflammation [58], whereas PE(15:0/24:1(15Z)) 20.934004, a phosphatidylethanolamine [59], is at low levels in platelets of obese patients [60]. Furthermore, a study highlighted the role of mitochondrial phosphatidylethanolamine in the regulation of uncoupling protein 1 (UCP1) to promote brown fat thermogenesis [61]. This suggests that CRD can control the onset and progression of obesity by affecting various beneficial metabolites.

Findings suggest that CRDs are able to modulate an imbalanced gut microbiota [62]. The number of Firmicutes in obese mice is higher than that of normal mice, whereas the quantity of Bacteroidota is lower in obese mice; these findings suggest that the gut microbes’ remodeling effect may play a role in the anti-obesity effect of CRDs [46]. Notably, Deng et al. observed reduced *Bacteroides* abundance at the genus level in the HFD group [62]. In contrast, our study revealed elevated Bacteroides levels in the HFD group, a finding supported by multiple investigations [11,47]. Notably, and in line with findings from several research studies [32,63,64], Bacteroidetes and Firmicutes were more abundant at the phylum level in all groups (Figure 5E). In line with the findings of Kang et al., we discovered that the abundance of *Akkermansia* was low in obese mice in our study, but increasing levels were observed after CRD therapy (Figure 5F), and these levels tended to be larger than the abundance of *Akkermansia* in normal mice [65]. And *Akkermansia* abundance demonstrated a negative correlation with obesity [66]. Furthermore, the dysregulation of gut microbiota in obese mice has been linked to *Dubosiella* imbalance, with an increase in *Dubosiella* levels partially restoring HFD-induced microbiota disruptions [67]. Our study demonstrated that CRD could effectively restore *Dubosiella* levels, suggesting that CRD’s obesity-alleviating effects may be mediated through gut microbiota regulation.

By Spearman’s correlation analysis, DL-2-aminooctanoic acid and Myo-Inositol were found to be negatively correlated with various obesity parameters including perirenal fat weight, liver weight, body weight, inguinal fat weight, and subcutaneous adipose tissue weight (Figure 7A, *p* < 0.05). On the other hand, 6-deoxyfagomine, LysoPC(16:0) Esi+18.909994, PC(18:1(9Z)/18:1(11Z)) Esi+22.989008, PC(18:1(9Z)/18:1(11Z)) Esi+23.05401, and LysoPC(0:0/18:0) Esi+ 20.252996 were positively correlated with the same obesity parameters (Figure 7A, *p* < 0.05). Myo-Inositol, as a sugar alcohol compound [68], has been shown to reduce body weight and regulate metabolism in obese individuals [49]. On the other hand, LysoPC(16:0) Esi+18.909994 is lysophosphatidylcholine. It has been shown that HDL cholesterol leads to elevated levels of lysophosphatidylcholine, which can serve as a key marker of obesity [69]. Kim et al. found that obese mice had higher levels of LysoPC compared to the dietary intervention group [70], aligning with the outcomes of our correlation analysis. PC(18:1(9Z)/18:1(11Z))Esi+22.989008 is a phosphatidylcholine whose elevated levels are associated with insulin resistance [71]. Elevated levels of phosphatidylcholine were observed in obese individuals, with a decrease noted following metformin intervention [72]. Our correlation analysis further supports a positive relationship between PC(18:1(9Z)/18:1(11Z)) Esi+22.989008 and obesity parameters, aligning with our research findings.

We conducted a correlation analysis between microorganisms and various obesity parameters, including perinephric fat weight, liver weight, body weight, inguinal fat weight, and subcutaneous adipose tissue weight (Figure 7B). This investigation aimed to identify which microorganisms might contribute to or combat obesity. Our findings indicated that *Incertae_Sedis*, *Colidextribacter*, *Oscillibacter*, *Candidatus_Saccharimonas*, and *Mucispirillum* exhibited strong positive correlations with all obesity parameters (*p* < 0.05), suggesting their potential involvement in obesity development. Conversely, *Parasutterella*, *Alloprevotella*, *Prevotellaceae*_NK3B31_group, *Alistipes*, unclassified_*Clostridia*_vadinBB60_group, and unclassified_*Muribaculaceae* showed strong negative correlations with obesity parameters, hinting at their potential inhibitory roles in obesity development (Figure 7B, *p* < 0.05).

One study reported that the *Incertae_Sedis* abundance was higher in HFD-induced obese mice, and dietary intervention reversed its level [73], which is consistent with our correlation analysis. Interestingly, it has also been reported that an increase in the number of *Incertae_Sedis* ameliorated obesity-induced metabolic disorders [74], but this is in contrast to our findings. Wang et al. demonstrated that reducing *Colidextribacter* numbers restored gut microbiota disruption induced by an HFD [75]. Probiotic treatment effectively decreased *Oscillibacter* abundance in the HFD group [76]. Similarly, lowering *Candidatus_Saccharimonas* levels was shown to modulate HFD-induced gut microbiota disruption in a study by Li et al. [77]. Studies have consistently reported an increase in *Mucispirillum* numbers with HFD consumption [78], corroborating our correlation analysis results.

We found that there was a negative link between obesity characteristics and *Parasutterella* in our study. It is interesting to note that, despite the negative link our results show, a number of other studies have found a positive correlation between obesity and *Parasutterella* [79,80,81]. On the other hand, it was also discovered that dietary intervention increased the levels of *Parasutterella*, which was shown to be less abundant in the HFD group than in the LFD group [82]. Prior studies have demonstrated the advantageous function of *Parasutterella* in preserving the homeostasis of bile acids [83], which aligns with our study findings. According to Li et al., the HFD group had a lower abundance of *Alloprevotella* than the LFD group, and medication therapy raised the levels of this organism [11]. Likewise, Lai et al. found that *Prevotellaceae*_NK3B31_group was negatively correlated with body weight, fat weight, and several harmful metabolites [84], aligning with our own research findings.

*Alistipes* was shown to be less abundant in the HFD group and more plentiful in the normal group in the study by Fu et al., with therapy raising its levels [85]. Additionally, it was demonstrated that unclassified_*Clostridia*_vadinBB60_group exhibited a negative correlation with obesity indicators [86]. Furthermore, unclassified_*Muribaculaceae*, as highlighted in the research by Kou et al., showed a lower abundance in the HFD group, and treatment led to an upregulation of its levels [82]. These findings collectively suggest that *Parasutterella*, *Alloprevotella*, *Prevotellaceae*_NK3B31_group, *Alistipes*, unclassified_*Clostridia*_vadinBB60_group, and unclassified_*Muribaculaceae* may collectively contribute to an anti-obesity role.

Although some of the results from the previously stated papers deviated from our findings, this discrepancy might be explained by the restricted generalizability resulting from the comparatively small number of obesity factors that were taken into account in our investigation. Nevertheless, summarily, our research underscores that CRD exhibits promise in combating obesity through enhancing metabolism and influencing the gut microbiota. Furthermore, DL-2-aminooctanoic acid and obesity showed a negative correlation in the correlation analysis, indicating that the compound may have an anti-obesity impact. However, there are currently no studies to support this, but perhaps in the future, academics will look into this.

## 5. Conclusions

According to our research, CRD has demonstrated the ability to reduce liver and colon tissue lesions, improve insulin sensitivity in high-fat conditions, and mitigate body weight and fat accumulation in HFD-induced obese mice. Furthermore, CRD shows promise in combating obesity through the regulation of endogenous metabolism, addressing microbiota imbalances, and maintaining intestinal flora stability by enhancing beneficial bacteria presence and reducing harmful bacteria levels. Our study also identified specific metabolites, such as inositol and 6-deoxyfagomine, which play a crucial role in ameliorating endogenous metabolic disorders as part of CRD’s beneficial effects. While these findings enhance our understanding of CRD’s impact on obesity, further research is necessary to elucidate the precise contributions of different metabolites and microorganisms to the development of obesity. Moreover, CRD holds promise as a natural remedy for addressing obesity and regulating dysbiosis. To gain a deeper understanding of its efficacy, conducting clinical trials is essential for assessing the long-term benefits of CRD on weight loss and the enhancement of the gut microbiota.

## Data Availability

Please contact the corresponding author for access to the data used in this study due to privacy.

## Supplementary Materials

The following supporting information can be downloaded at https://www.mdpi.com/article/10.3390/nu16172859/s1; Figure S1. Heat maps and enrichment bubble maps of metabolites. (A) Heat map displaying the four experimental groups’ respective metabolite intensities, (B) metabolic pathways enriched in metabolites. Figure S2. LEfse analysis of intestinal flora. (A) Microorganisms with LDA scores greater than 4 were categorized, (B) Evolutionary branching diagram based on LEfse analysis.
