# Supplementary material for: Cordycepin Ameliorates High Fat Diet-Induced Obesity by Modulating Endogenous Metabolism and Gut Microbiota Dysbiosis
Source: Nutrients. 2024 Aug 27;16(17):2859. doi: 10.3390/nu16172859 (PMC11396883; doi:10.3390/nu16172859)
Supplement: Supplementary file 1 [file nutrients-16-02859-s001.zip › nutrients-3133286-supplementary.pdf]

A

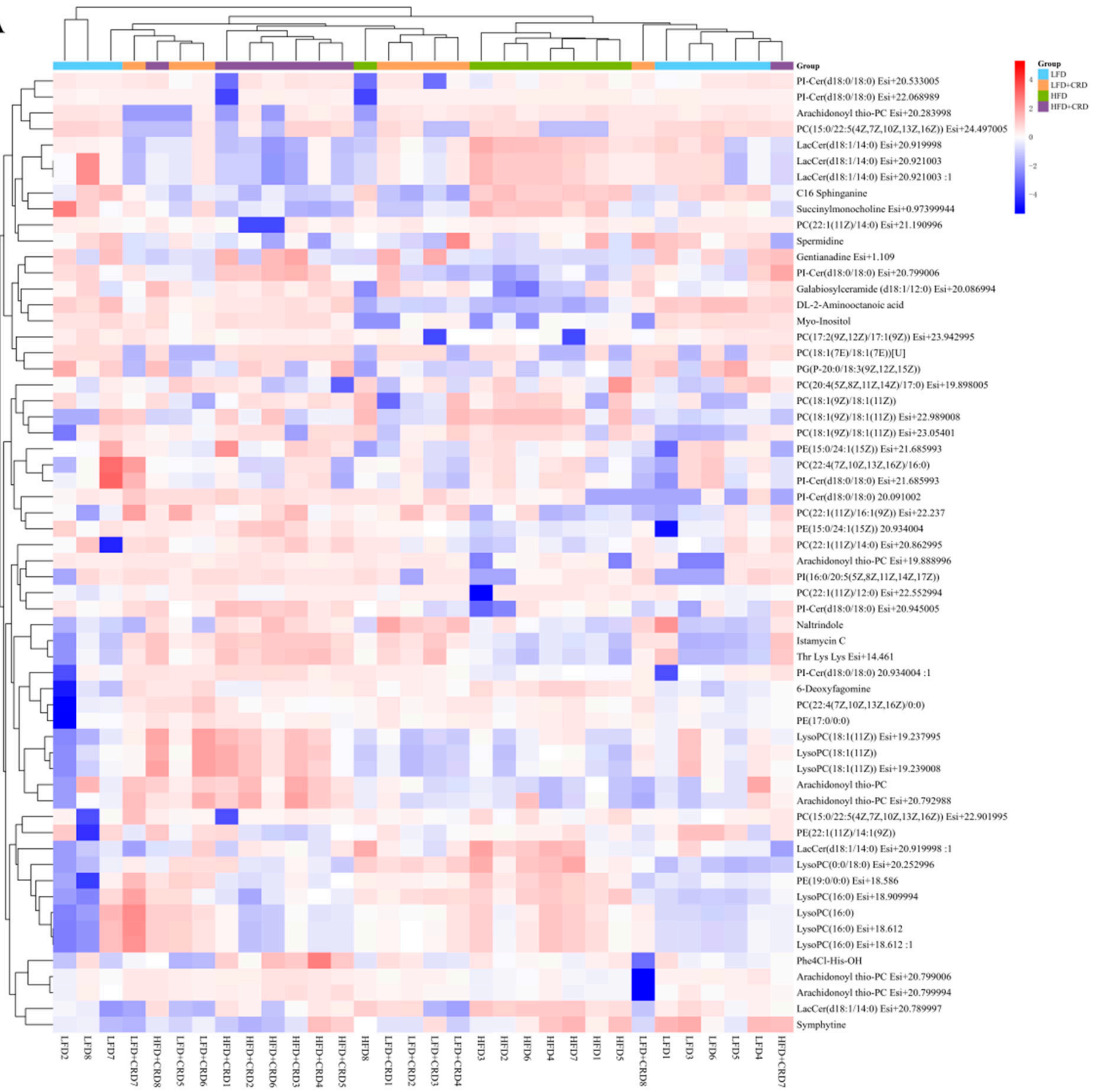

Group
Group
LFD
LFD+CRD
HFD
HFD+CRD
4
2
0
-2
-4
PI-Cer(d18:0/18:0) Esi+20.533005
PI-Cer(d18:0/18:0) Esi+22.068989
Arachidonoyl thio-PC Esi+20.283998
PC(15:0/22:5(4Z,7Z,10Z,13Z,16Z)) Esi+24.497005
LacCer(d18:1/14:0) Esi+20.919998
LacCer(d18:1/14:0) Esi+20.921003
LacCer(d18:1/14:0) Esi+20.921003 :1
C16 Sphinganine
Succinylmonocholine Esi+0.97399944
PC(22:1(11Z)/14:0) Esi+21.190996
Spermidine
Gentianadine Esi+1.109
PI-Cer(d18:0/18:0) Esi+20.799006
Galabiosylceramide (d18:1/12:0) Esi+20.086994
DL-2-Aminooctanoic acid
Myo-Inositol
PC(17:2(9Z,12Z)/17:1(9Z)) Esi+23.942995
PC(18:1(7E)/18:1(7E))[U]
PG(P-20:0/18:3(9Z,12Z,15Z))
PC(20:4(5Z,8Z,11Z,14Z)/17:0) Esi+19.898005
PC(18:1(9Z)/18:1(11Z))
PC(18:1(9Z)/18:1(11Z)) Esi+22.989008
PC(18:1(9Z)/18:1(11Z)) Esi+23.05401
PE(15:0/24:1(15Z)) Esi+21.685993
PC(22:4(7Z,10Z,13Z,16Z)/16:0)
PI-Cer(d18:0/18:0) Esi+21.685993
PI-Cer(d18:0/18:0) 20.091002
PC(22:1(11Z)/16:1(9Z)) Esi+22.237
PE(15:0/24:1(15Z)) 20.934004
PC(22:1(11Z)/14:0) Esi+20.862995
Arachidonoyl thio-PC Esi+19.888996
PI(16:0/20:5(5Z,8Z,11Z,14Z,17Z))
PC(22:1(11Z)/12:0) Esi+22.552994
PI-Cer(d18:0/18:0) Esi+20.945005
Naltrindole
Istamycin C
Thr Lys Lys Esi+14.461
PI-Cer(d18:0/18:0) 20.934004 :1
6-Deoxyfagomine
PC(22:4(7Z,10Z,13Z,16Z)/0:0)
PE(17:0/0:0)
LysoPC(18:1(11Z)) Esi+19.237995
LysoPC(18:1(11Z))
LysoPC(18:1(11Z)) Esi+19.239008
Arachidonoyl thio-PC
Arachidonoyl thio-PC Esi+20.792988
PC(15:0/22:5(4Z,7Z,10Z,13Z,16Z)) Esi+22.901995
PE(22:1(11Z)/14:1(9Z))
LacCer(d18:1/14:0) Esi+20.919998 :1
LysoPC(0:0/18:0) Esi+20.252996
PE(19:0/0:0) Esi+18.586
LysoPC(16:0) Esi+18.909994
LysoPC(16:0)
LysoPC(16:0) Esi+18.612
LysoPC(16:0) Esi+18.612 :1
Phe4Cl-His-OH
Arachidonoyl thio-PC Esi+20.799006
Arachidonoyl thio-PC Esi+20.799994
LacCer(d18:1/14:0) Esi+20.789997
Symphytine
LFD2
LFD8
LFD7
LFD+CRD7
HFD+CRD8
LFD+CRD5
LFD+CRD6
HFD+CRD1
HFD+CRD2
HFD+CRD6
HFD+CRD3
HFD+CRD4
HFD+CRD5
HFD8
LFD+CRD1
LFD+CRD2
LFD+CRD3
LFD+CRD4
HFD3
HFD2
HFD6
HFD4
HFD7
HFD1
HFD5
LFD+CRD8
LFD1
LFD3
LFD6
LFD5
LFD4
HFD+CRD7

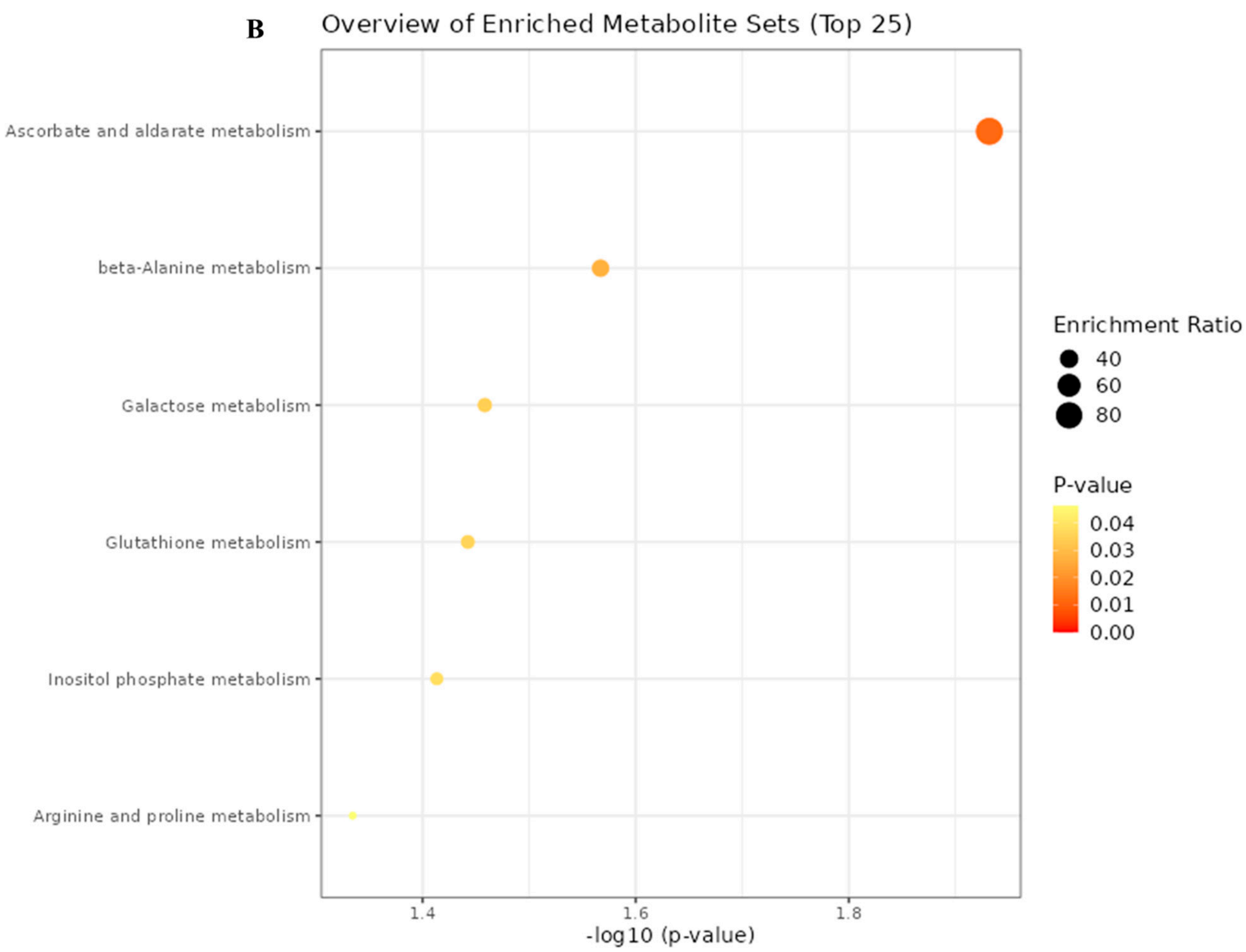

Supplementary Figure S1. Heat maps and enrichment bubble maps of metabolites. (A) Heat map displaying the four experimental groups' respective metabolite intensities, (B) metabolic pathways enriched in metabolites.

A

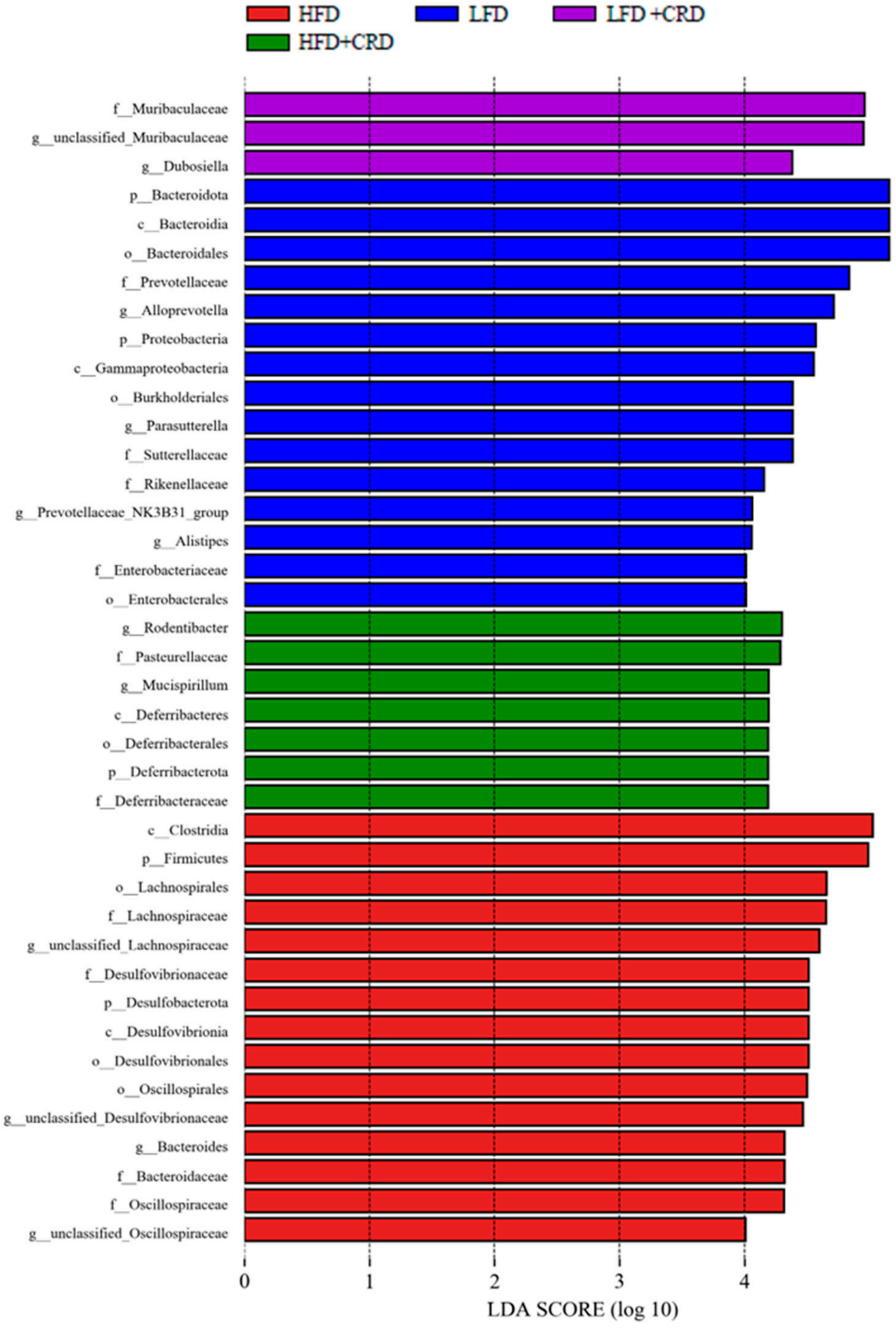
HFD
LFD
LFD +CRD
HFD+CRD
f__Muribaculaceae
g__unclassified_Muribaculaceae
g__Dubosiella
p__Bacteroidota
c__Bacteroidia
o__Bacteroidales
f__Prevotellaceae
g__Alloprevotella
p__Proteobacteria
c__Gammaproteobacteria
o__Burkholderiales
g__Parasutterella
f__Sutterellaceae
f__Rikenellaceae
g__Prevotellaceae_NK3B31_group
g__Alistipes
f__Enterobacteriaceae
o__Enterobacterales
g__Rodentibacter
f__Pasteurellaceae
g__Mucispirillum
c__Deferribacteres
o__Deferribacterales
p__Deferribacterota
f__Deferribacteraceae
c__Clostridia
p__Firmicutes
o__Lachnospirales
f__Lachnospiraceae
g__unclassified_Lachnospiraceae
f__Desulfovibrionaceae
p__Desulfobacterota
c__Desulfovibrionia
o__Desulfovibrionales
o__Oscillospirales
g__unclassified_Desulfovibrionaceae
g__Bacteroides
f__Bacteroidaceae
f__Oscillospiraceae
g__unclassified_Oscillospiraceae
0
1
2
3
4
LDA SCORE (log 10)

B

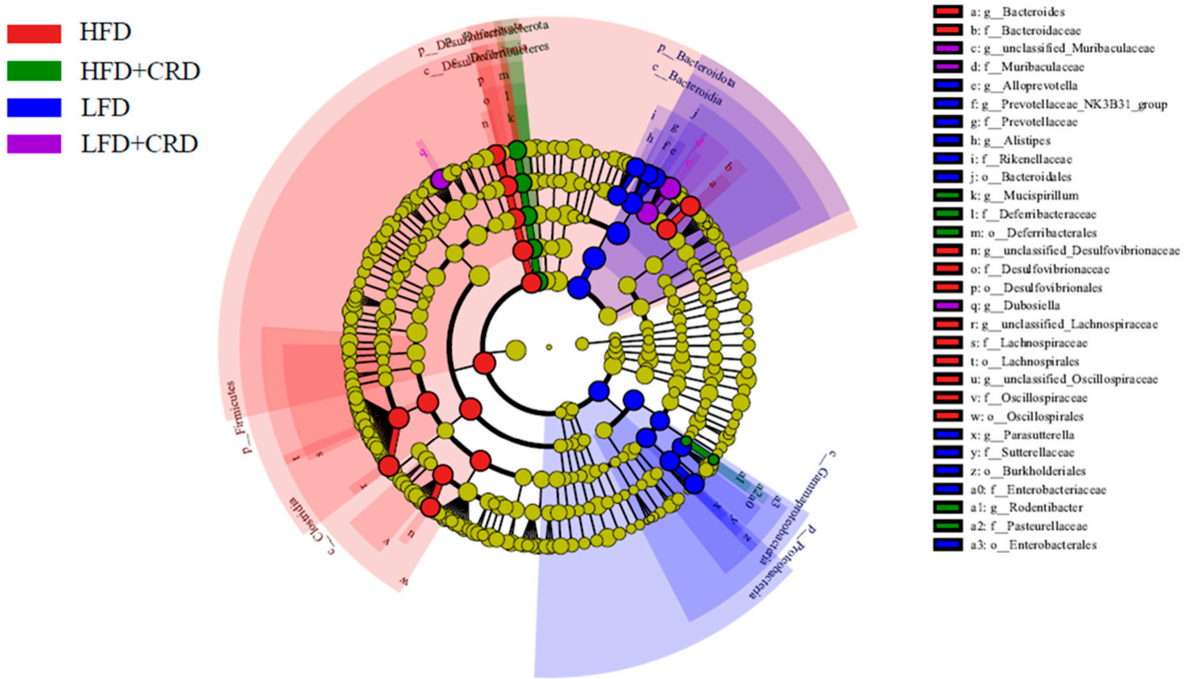

Supplementary Figure S2. LEfse analysis of intestinal flora. (A) Microorganisms with LDA scores greater than 4 were categorized, (B) Evolutionary branching diagram based on LEfse analysis.
